# Machine Learning and Internet of Things Enabled Monitoring of Post-Surgery Patients: A Pilot Study

**DOI:** 10.3390/s22041420

**Published:** 2022-02-12

**Authors:** Saeed Ali Alsareii, Mohsin Raza, Abdulrahman Manaa Alamri, Mansour Yousef AlAsmari, Muhammad Irfan, Umar Khan, Muhammad Awais

**Affiliations:** 1 Department of Surgery, College of Medicine, Najran University Saudi Arabia, Najran 11001, Saudi Arabia; amsalamri@nu.edu.sa (A.M.A.); myalasmari@nu.edu.sa (M.Y.A.); 2 Department of Computer Science, Edge Hill University, St Helens Rd., Ormskirk L39 4QP, UK; razam@edgehill.ac.uk (M.R.); khanu@edgehill.ac.uk (U.K.); 3 Electrical Engineering Department, College of Engineering, Najran University, Najran 11001, Saudi Arabia; miditta@nu.edu.sa

**Keywords:** Internet of Things (IoT), machine learning (ML), Artificial Intelligence (AI), healthcare, patient monitoring, human activity classification (HAC), obesity, ultra-reliable low latency communication (URLLC), gradient boosting regression, post-surgery recovery

## Abstract

Artificial Intelligence (AI) and Internet of Things (IoT) offer immense potential to transform conventional healthcare systems. The IoT and AI enabled smart systems can play a key role in driving the future of smart healthcare. Remote monitoring of critical and non-critical patients is one such field which can leverage the benefits of IoT and machine learning techniques. While some work has been done in developing paradigms to establish effective and reliable communications, there is still great potential to utilize optimized IoT network and machine learning technique to improve the overall performance of the communication systems, thus enabling fool-proof systems. This study develops a novel IoT framework to offer ultra-reliable low latency communications to monitor post-surgery patients. The work considers both critical and non-critical patients and is balanced between these to offer optimal performance for the desired outcomes. In addition, machine learning based regression analysis of patients’ sensory data is performed to obtain highly accurate predictions of the patients’ sensory data (patients’ vitals), which enables highly accurate virtual observers to predict the data in case of communication failures. The performance analysis of the proposed IoT based vital signs monitoring system for the post-surgery patients offers reduced delay and packet loss in comparison to IEEE low latency deterministic networks. The gradient boosting regression analysis also gives a highly accurate prediction for slow as well as rapidly varying sensors for vital sign monitoring.

## 1. Introduction

Over the past two years, the healthcare sector has been severely affected. The COVID-19 outbreak has impaired the healthcare infrastructure and introduced many unthinkable challenges to the present healthcare ecosystem. The high strain on the healthcare sector has resulted in general degradation of overall healthcare services with the non-critical departments being most influenced due to the focused utilization of limited resources and qualified healthcare staff. One such aspect which has been most affected during the COVID-19 outbreak is post-surgery care and therefore, needs drastic measures to restore the healthcare standards. This is specifically needed in the less critical healthcare sectors where the dire need of continuous monitoring is evident but cannot be ensured due to lack of healthcare staff.

The aftercare in a post-surgery phase is an important aspect of any surgery. The recovery of the patient depends on the effective execution of aftercare in the post-surgery phase. Although the patients in some surgeries can leave hospital in 3 to 5 days, the return to normal activities may take much longer. For instance, in weight loss surgeries, the patients can leave hospital within 1 to 3 days, but it takes 4 to 6 weeks to return to routine life and even then, the patients are expected to follow a relatively strict routine of exercise and maintain suitable eating habits [1,2]. The regular follow-up appointments may last for two years [3]. Therefore, it is important to provide necessary care to the patients in the post-surgery phase and allocate appropriate resources to support their healthy recovery. Unfortunately, this puts an additional load on the already strained healthcare system. Especially, in the COVID-19 pandemic, it is becoming difficult to allocate enough healthcare staff to deal with all healthcare contingencies. It is also noteworthy that the added load on healthcare services has delayed non-crucial surgeries, where most of the support has been diverted to more life threating and critical cases.

While the healthcare services have suffered in the past few years due to COVID-19, suitable technological interventions have also surfaced to mitigate the impact of such a health crisis and limit its devastating effects on the healthcare infrastructure. New and innovative technology solutions are needed to cope with the overwhelming load on the healthcare sector. The use of machine learning techniques and Internet of Things (IoT) has enabled smart healthcare systems to offer potential solutions to address surgery related and aftercare problems.

This paper presents an IoT enabled and machine learning driven solution to monitor the vitals of the critical patients while focusing on post-surgery patients. In post-surgery patients monitoring of the cardiovascular systems, fluid and electrolyte balance, prevalence and treatment of infection and excessive bleeding, functioning of main organs, deep vein thrombosis, and anastomotic leak need to be monitored with high reliability and relatively low latency. Therefore, the proposed framework focuses on IoT enabled ultra-reliable low latency communications (URLLC) and machine learning based change in vitals prediction. The main contributions of the work are as follows:

1. An IoT enabled URLLC framework is proposed which offers highly reliable near real-time communication of the critical patients’ vitals. It also allows simultaneous channel access for critical and non-critical patients and ensures prioritized access to critical patients.
2. The paper also proposes to use a gradient boosting based regression algorithm capable of predicting both slow and rapidly changing patient’s vitals. The proposed algorithm serves as an additional layer of protection for critical patients, where in the event of wireless communications failure, the ML algorithm will still be able to predict the patient’s vitals, thus raising alarms even if the wireless channel faces intermittent interference or sudden variations resulting in communication failure.

## 2. Literature Review

The IoT enabled remote monitoring and smart healthcare systems offer great potential to cope with the challenges in healthcare services [4,5,6]. The IoT enabled smart systems have applications in almost all aspects of healthcare and have the potential to resolve many challenges and ease the load on healthcare workers and qualified medical staff. With the help of IoT and machine intelligence, new and refined monitoring and diagnosis systems can be developed. One of the many application areas where IoT offers new and innovative solutions is post-surgery patient monitoring [7]. In most cases, patient monitoring is non-critical in nature, apart from a few selective cases. At the same time, it is highly time and resource consuming to offer suitable healthcare services to such patients [8]. IoT provides the infrastructure to enable remote monitoring of such patients more effectively with less resources. With the vitals from a patient taken every few seconds, and an alerting system in place to detect any anomalies, IoT can provide a reliable and highly effective solution for the post-surgery care and recovery of the patients.

The IoT serves as an excellent solution with great potential to accumulate data from different sensors. However, there are certain limitations which need undue attention to make the system reliable and infallible when dealing with patients. While there are several works which address some challenges in IoT to make them more suitable for healthcare applications [9,10,11,12], these works suffer from many limitations in terms of resource allocation and prioritization of vulnerable patient communications. One such limitation is the allocation of the appropriate channel resources to individual surgery patients to communicate their vitals to the qualified staff with low latency and high reliability. It becomes more challenging when the resource requirements from different surgery patients in a ward differ from others and with some being more critical than others. Sangiah et al. [13] developed an IoT system for resource allocation in healthcare using the whale optimization algorithm. However, the system provided a generic solution for the resource allocation and did not include a healthcare infrastructure as an application scenario. Similarly, Baker et al. [14] proposed a system and used the ideology of naming everything as a resource, implemented in the generic healthcare scenario. The analyzed features were capacity (computational, consumed resources) and the resource allocation in terms of restriction (who can and cannot use the resources). However, the work was not focused on an automatic resource allocation mechanism and did not propose any in-hospital paradigm to enable priority communication through monitoring the patients’ vital signs. The authors in [15] suggested that service orchestration and service management are still challenging issues in healthcare applications and services. Moreover, the existing systems do not possess the capability to handle the requests and services required locally by the healthcare infrastructure. Another challenge is that the existing systems [11,16,17] have mainly focused on generic healthcare applications and focused little on developing IoT systems for post-surgery patients to monitor their vital signs and recovery patterns. Post-surgery monitoring mainly involves monitoring of the cardiovascular systems, regular functioning of the main organs, fluid and electrolyte balance, prevalence and treatment of infection and excessive bleeding, deep vein thrombosis, anastomotic leak, dietary requirements, and progression [18]. Therefore, a suitable IoT framework is needed to enable communications from diverse surgery patients in a timely fashion. It is also important that the proposed framework bears the potential to cope with long-distance monitoring of the patients once they are discharged from hospital.

Considering the key challenges in monitoring surgery patients during short hospital-based aftercare and extended monitoring in home environments, an IoT framework is proposed which not only retains consideration of the reliability and latency aspects of wireless communications but also provides a decision support system to enable quick hassle-free decision making for the medical staff. The proposed work not only saves time and medical resources but also offers more reliable and actionable information to implement improved healthcare services for the speedy recovery of surgery patients.

The rest of the paper is organized as follows: A system model is presented in Section 2, results and discussion are provided in Section 3. Finally, the concluding remarks are displayed in Section 4.

## 3. System Model and Methods

Over the past few years, IoT has evolved to incorporate a plethora of technologies including ubiquitous sensing, fog computing, big data, machine intelligence, decision support systems, cyber physical systems, distributed systems, edge computing, diagnostic systems, process automation, etc. Thus, the present day IoT enabled smart systems serve as the convergence of key enabling technologies capable of handling diverse applications with the desired precision and accuracy. With the rapidly increasing number of connected devices, and the evolution of microelectromechanical systems, the availability of big data and the ability of machine intelligence have enabled the emergence of true AI. The IoT infrastructure necessary to sample the real-world with the communication of sensory data whether it is the healthcare sector, industry, buildings or vehicles, is essential for sustainable future developments. To meet the challenges of big data and collective intelligence, effective IoT solutions play a significant role.

The proposed IoT framework aims to provide Ultra-Reliable Low Latency Communication (URLLC) [19] in healthcare and mitigate uncertainty in critical communications. Further details of the system parameters are covered in the subsequent discussion.

### 3.1. IoT Topology and Superframe Structure

**Figure 1:**
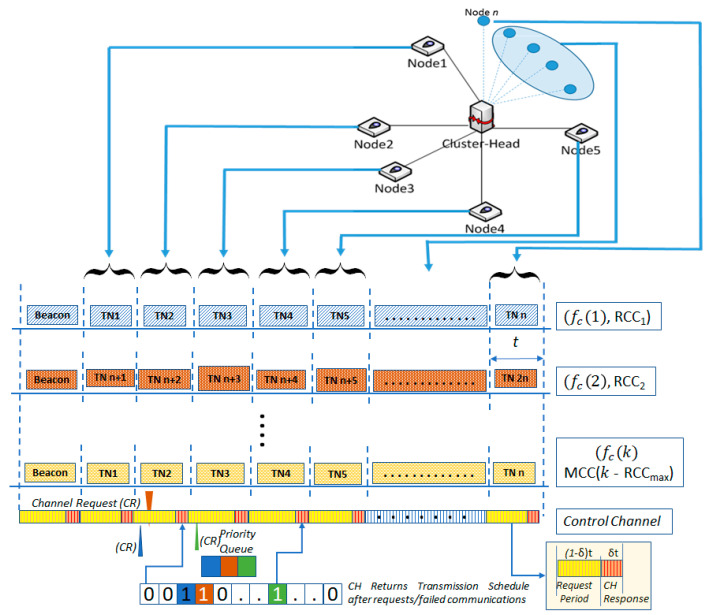
Superframe structure and network topology.

In the proposed work, the communications from the sensor motes are established in a cluster, where hierarchical architecture is assumed for reduced delays and scalability. In each cluster, a star topology is formed, where communications from cluster nodes to cluster-head (CH) are facilitated by time division multiple access (TDMA) based channel segmentation. The cluster formation is initiated by the designated CH in setup mode, where IoT devices/sensor nodes request affiliation to one of the available CHs based on its geographic location and link quality. The selection of TDMA over more conventional Carrier Sense Multiple Access, Collision Avoidance (CSMA/CA) is preferred to avoid congestion in the network and enable guaranteed channel access in a specific timeslot for the communicating node. Despite the use of TDMA as the multiple access technique, suitable changes have been introduced to improve the overall accessibility of IoT devices and prioritize critical information to maintain low latency communications within the network. The communications from sensing nodes to CH takes place in a TDMA based superframe of duration T. Each superframe is further divided into n timeslots each of duration, t, where each timeslot allows communication from one sensor node. A control channel is designated to manage the rescheduling of failed communications. The proposed IoT infrastructure is presented in Figure 1, whereas the frequently used system parameters are listed in Table 1. These parameters are used to evaluate the performance of the proposed scheme in the MATLAB software.

### 3.2. Multi-Channel Scenario and Communication Rescheduling

As presented in Figure 1, the proposed IoT framework makes use of the star topology in which CH evaluates affiliation requests from the nodes to decide the node’s suitability to associate with this cluster. Once the nodes are affiliated to the relevant clusters, each node is issued a local id between 1 to w, where w represent the maximum nodes/motes within a cluster. It is worth mentioning that while the cluster represented in the figure corresponds to a single channel, a multi-channel scheme is introduced.

All the communication channels are distributed among HPC and MCC, where communication on each channel uses the TDMA based scheme to avoid collisions and channel reliability opposed to carrier sense multiple access (CSMA) schemes. In a single channel scenario for IEEE low latency deterministic networks (LLDN), the probability of at least one failed communication in a superframe is modelled using binomial distribution [20,21] and can be expressed as

(1) $$P\left(comm.\;failure\right)=\frac{n!}{1!\left(n-1\right)!}p{\left(1-p\right)}^{n-1}+\frac{n!}{2!\left(n-2\right)!}p^{2}(1-\;p{)}^{n-2}+\ldots +\frac{n!}{\left(n-1\right)!\left(n-\left(n-1\right)\right)!}p^{n-1}{\left(1-p\right)}^{n-\left(n-1\right)}+\frac{n!}{\left(n\right)!\left(n-\left(n\right)\right)!}p^{n}{\left(1-p\right)}^{n-n}$$

Taking into consideration the multi-channel scenario where each superframe communication at channel, $f_{c}\left(i\right)$ is assumed to be independent of superframe communication at channel $f_{c}\left(j\right)$, where i≠j the communication failure in any superframe is given by

(2) $$P\left(Avg.\;comm.\;failure\;\right)=\underset{f_{C}\left(i\right)\in F_{c}}{\sum }\frac{1}{k}\times \left(\overset{n}{\underset{x=1}{\sum }}\left(\begin{array}{c} n\\ x \end{array}\right)p_{i}^{x}{\left(1-p_{i}\right)}^{n-x}\right)$$

where $F_{c}$ is the number of frequency channels, k channels are used in the superframe and $p_{i}$ is the communication failure probability for the channel $f_{C}\left(i\right)$. The average communication failure is normalized with the number of channels which is *k*, as expressed in Equation (2). All channel communications are synchronized, where a synchronization beacon is issued at the start of every superframe. In addition, a control channel is also introduced, which allows the IoT devices belonging to high priority/vulnerable patients/systems to either request communication resource or CH reschedules their retransmission as needed. However, a more frequent practice, where the CH automatically reschedules communications of high priority nodes, is considered for evaluation purposes.

The nodes communicating over HPC have higher priority compared to the nodes communicating over MCC. In addition, the sensory data accumulated through the HPC belong to critical/vulnerable patients which are time sensitive and need to be communicated to CHs in regular intervals. A failure/delay in such communications can put the critical patients at risk. Therefore, the deviation from regular delay intervals needs to be maintained within certain thresholds.

Under normal circumstances the communications from TDMA based HPC and MCC continue uninterruptedly. In case of failure in communication from one of the sensor nodes in HPC, i.e., the high priority nodes, the CH reschedules a communication on MCC, where failed communication is reattempted within the same superframe. The communication failure of an HPC superframe can be expressed as

(3) $$\begin{array}{ll} P(comm.\;failure & \;in\;HPC\;superframe)\\ & =\frac{n!}{1!\left(n-1\right)!}n{\left(1-n\right)}^{n-1}\left(p\right)\\ & +\frac{n!}{2!\left(n-2\right)!}p^{2}(1\\ & -p{)}^{n-2}[\overset{2}{\underset{x=1}{\sum }}\left(\begin{array}{c} 2\\ x \end{array}\right)p^{x}(1\\ & -p{)}^{2-x}]+\ldots +\frac{n!}{\left(n-1\right)!\left(n-\left(n-1\right)\right)!}p^{n-1}(1\\ & -p{)}^{n-\left(n-1\right)}\left[\overset{n-1}{\underset{x=1}{\sum }}\left(\begin{array}{c} n-1\\ x \end{array}\right)p^{x}{\left(1-p\right)}^{n-1-x}\right]\\ & +\frac{n!}{\left(n\right)!\left(n-\left(n\right)\right)!}p^{n}{\left(1-p\right)}^{n-n}\left[\overset{m}{\underset{x=1}{\sum }}\left(\begin{array}{c} m\\ x \end{array}\right)p^{x}{\left(1-p\right)}^{m-x}\right] \end{array}$$

In the case of HPC less than or equal to MCC, i.e., $R_{s}\leq M_{s}$, the above equation can be approximated to

(4) $$\left(comm.\;failure\;in\;RCC\;superframe\;\forall \;R_{s}\leq M_{s}\right)\;\;\;\;\;=\overset{m}{\underset{y=1}{\sum }}\left[\left(\left(\begin{array}{c} n\\ y \end{array}\right)p^{y}{\left(1-p\right)}^{n-y}\right)\left(\overset{y}{\underset{x=1}{\sum }}\left(\begin{array}{c} z\\ x \end{array}\right)p^{x}{\left(1-p\right)}^{z-x}\right)\right]\;$$

As expressed in Equations (3) and (4), binomial distribution is primarily used to express the failures in the superframe communications. The communication rescheduling allows a timely access to the channel, thus minimizing the average delay along with improving system reliability.

Traditional applications accept a relatively high average variation from specified delay intervals between two consecutive communications. However, in the case of critical healthcare applications, deviation from a defined interval can severely elevate the health risks in vulnerable patients and thus, limit the effectiveness of IoT enabled smart healthcare over conventional systems. Therefore, the delay in the proposed scheme is modelled as the average variation from the expected interval. In IEEE LLDN, the average deviation from the mean delay in HPC can be approximated as

(5) $$d_{LLDN}=\overset{n}{\underset{x=1}{\sum }}T\times \left(\begin{array}{c} n\\ x \end{array}\right)p^{x}{\left(1-p\right)}^{n-x}$$

In the proposed scheme the delay is minimized with rescheduling of the failed HPC data communication. The average deviation in HPC superframe communications from the specified delay time interval can be expressed as

(6) $$d_{HPC}=2t\times \overset{n-2}{\underset{x=1}{\sum }}\left(\begin{array}{c} n-2\\ x \end{array}\right)p^{x}{\left(1-p\right)}^{n-2-x}+5t\overset{n}{\underset{x=n-2}{\sum }}\left(\begin{array}{c} n\\ x \end{array}\right)p^{x}{\left(1-p\right)}^{n-x}$$

while the proposed IoT framework offers an improved performance in both delay and reliability, it is still vulnerable to diverse channel fading and interference. Therefore, in the following section, an AI based sensory data prediction system is modelled. Further details on the performance of the proposed work can be seen in the results and discussion section.

### 3.3. Predictive Modelling of Vitals for Surgery Patients

Surgery patients are expected to be monitored over the course of recovery, whether body sensors or other monitoring techniques are used. In this work, along with the effective communication network to relay the patient’s vitals using body sensors and IoT infrastructure, predictive analysis and modelling are also performed. In a particular scenario, where the communication fails for a short period of time or excessive interference develops which causes brief communication failure, the communication of vitals becomes impossible. Therefore, to avoid such scenarios where the instantaneous communication failure can obstruct the patients’ vitals to be delivered, a predictor is developed to counter the issue. The predictor uses the existing trail of sensory data received from the IoT devices to predict the future values of slow as well as rapidly varying sensors to generate the appropriate response. The prediction algorithm runs on the CH and offers predictive analysis of the sensory data of critical patients. Since the vital signatures (blood pressure, temperature, oxygen levels, etc.) of a patient can vary quickly [22], it is important to introduce machine learning techniques to predict the vitals (sensor values) and prioritize the patients in case the values approach close to the threshold. This achieves two purposes: (1) adaptive prediction of the vitals of a patient, thus ensuring continuous wellbeing, (2) a redundant monitoring mechanism in case the IoT based solution fails to function for a short duration or the wireless channel faces high interference and thus, higher than usual packet loss.

To achieve the prediction, two different scenarios are considered, where the patients’ sensory data are varied both linearly and exponentially to emphasize the possibility of slow change and rapid change in the patient’s condition. Further details on each of the machine learning techniques used for the predictive analysis are presented in the subsequent sections.

#### 3.3.1. Sensory Dataset for Vital Sign Monitoring

One of the significant issues in developing intelligent systems for clinical healthcare is the unavailability of the vital signs based sensory dataset and the clinical dataset recorded from the patients during in-hospital treatment. Such scenarios require a longitudinal study to follow up and monitor the pre-surgery and post-surgery vitals as in the quantitative dataset about the medical condition being treated, such as obesity in the case of bariatric surgery. Tracking and monitoring of such clinical and sensory measures are vital in predicting the recovery curve and rehabilitation progress. To run the pilot study, significant resources (time, financial support, infrastructural support) and ethical approvals are required from the healthcare providers. However, the development and acquisition of healthcare data from such scenarios in a short period of time is infeasible. Therefore, we generated synthetic sensory data simulating the real-life sensory traces/signals and tested the performance of the machine learning algorithm as a proof of concept. The synthetic data generated in this study are also not related to the accuracy of the data generation. Rather the study focuses on the change in the vitals’ monitoring which can be slow or rapid depending on patients’ health conditions. The aim is to justify that the proposed work can predict rapidly changing data with high accuracy. Therefore, after this proof-of-concept study, the aim is to develop a complete IoT based paradigm which can record and monitor healthcare vitals in post-operative in-hospital scenarios.

#### 3.3.2. Gradient Boosting Based Regression

To detect the vital sensory based indicators of patients, regression-based machine learning is used. In particular, this work developed an eXtreme Gradient Based (XGB) [23,24] regression model to monitor the vital signs. The XGB [25] is widely used in IoT based healthcare applications to objectively quantify complex patterns from healthcare-based sensors. The parameters used for XGB regression are as follows; booster = GB tree, minimum child weight = 1, maximum depth = 6, gamma = 0, subsample = 1, lambda = 1, and alpha = 0. The XGB is implemented in Python. Further details on sensory data and predictive analysis are covered in the results and discussion.

A 70/30 train and test split were used, i.e., 70% of the dataset was used to train the XGB regressor model and the remaining 30% to test the model.

## 4. Results and Discussion

**Figure 2:**
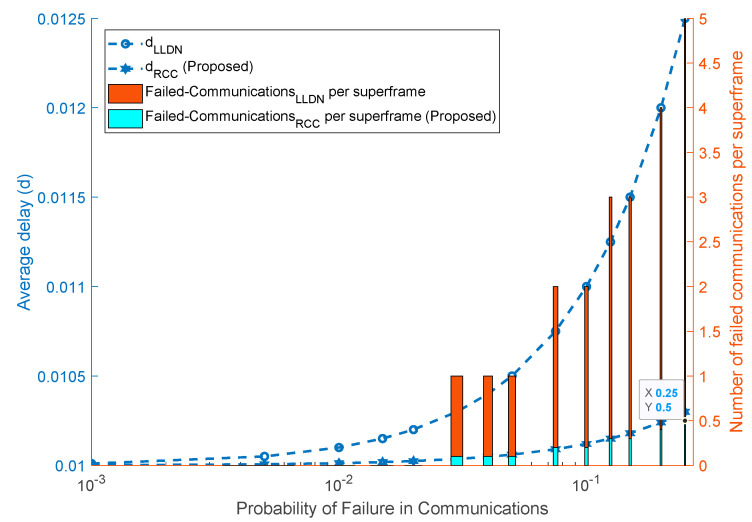
Average delay and communications reliability (RCC = 1, MCC = 3).

The performance of the proposed IoT framework is evaluated for the average delay and the reliability of communications. Since the proposed scheme facilitates periodic communications, delay and reliability are appropriate performance metrics for evaluation of the scheme. Figure 2 presents the results across two y-axes, left and right. The left Y-axis shows the average delay. The results presented in Figure 2 show that the proposed scheme reduces the overall delay by a significant margin. The deviation from the specified delay interval also lies within 1 ms in the case of the proposed IoT framework. In addition, the number of failed communications per superframe in the case of IEEE LLDN [26] is much higher compared to the proposed scheme. As a trade-off, in the proposed scheme, the performance of the monitoring systems is sacrificed, which is an acceptable trade-off for a mix of critical and non-critical applications.

The proposed work shows notable improvements in terms of delay where the average delay in the proposed work is significantly reduced compared to IEEE LLDN. The delay in the proposed scheme ($d_{\mathrm{RCC}}$) is reduced to 1/5th compared to IEEE LLDN (as presented in Figure 2), thus, providing a more efficient solution for critical health patients. In the proposed scheme, the failures in critical communications are also reduced notably in comparison to IEEE LLDN. The proposed scheme offers less failures ranging from 1% to 95% for different channel conditions. The overall improvement is depicted by bar graphs presented in Figure 2. The improvements in both delay and communication reliability are achieved at the cost of deteriorated performance in the monitoring communications which are non-critical data communications and thus present no notable harm. The relaxed deadlines in monitoring communications also reduce the impact of slight additional delays added in the monitoring services.

The proposed IoT framework although offers notable improvements in enabling effective communications, however, it cannot remedy the communication failures occurring due to intermittent interference. Therefore, it is equally important to leverage the use of machine learning techniques to enable predictive analysis of the sensory data collected from the patients using IoT. To evaluate the suitability of machine learning techniques and to predict the changes in sensory data, both slow and fast changing sensory data are considered. The performance of a gradient boosting regressor is evaluated for both linearly and exponentially varying sensory data. Further details on the suitability of machine learning algorithms are presented in subsequent paragraphs.

**Figure 3:**
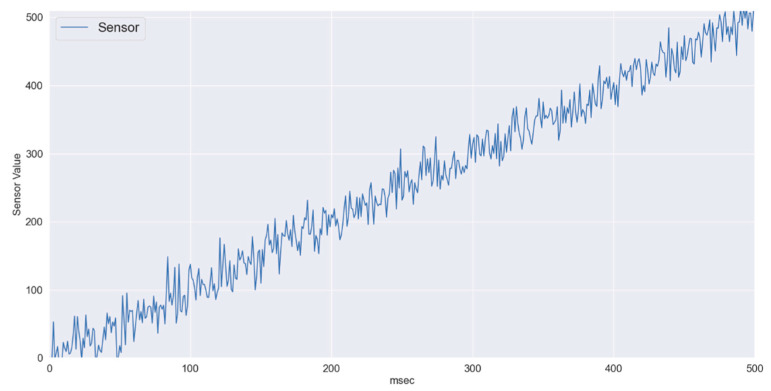
Slow changing sensory data.

**Figure 4:**
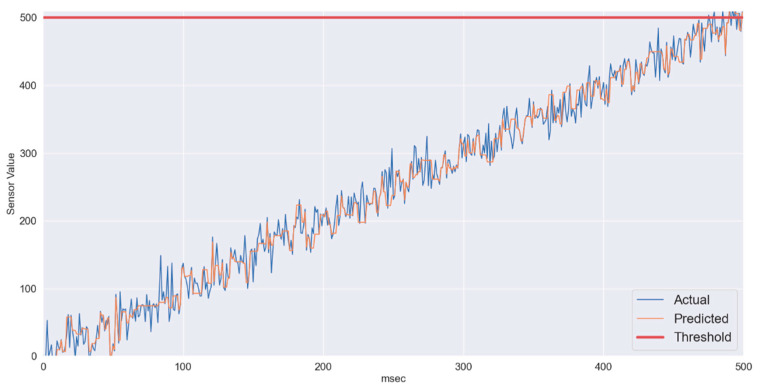
XGB-slow varying data: Predicted vs. Actual.

Figure 3 shows linearly changing sensory data. In periodically reported patient’s vitals using IoT, at first relatively slow changing data were considered where the change from normal sensory input to critical sensory input takes around 500 ms. In the IoT framework with superframe duration of 10 ms, over 50 samples from the sensor are expected to be communicated to the CH. Even if the wireless channel becomes highly unstable, the proposed gradient boosting regression running at the CH manages to predict the values with sufficient accuracy. In Figure 4, three series are presented, i.e., actual values or ground truth labels, the predicted labels obtained through the XGB classifier, and the threshold. The threshold presented identifies the critical threshold values for the sensor readings and if exceeded represents the equivalence of medical emergency. It is to illustrate how quickly the system will be able to detect/predict patients’ sensory traces violating the threshold. The mean absolute error obtained for this scenario is 3.47.

**Figure 5:**
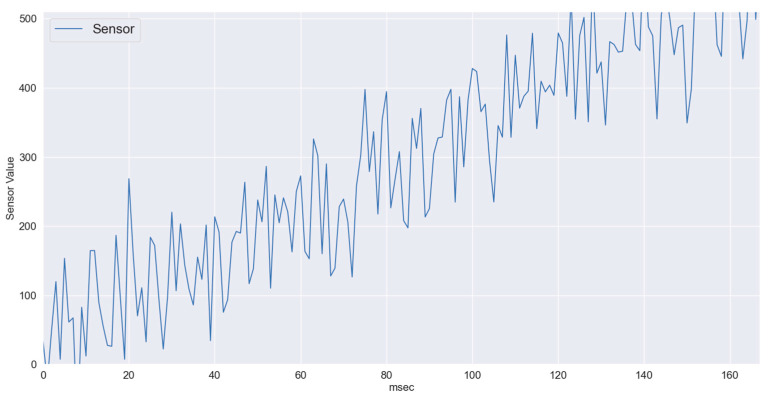
Varying Sensory Data.

**Figure 6:**
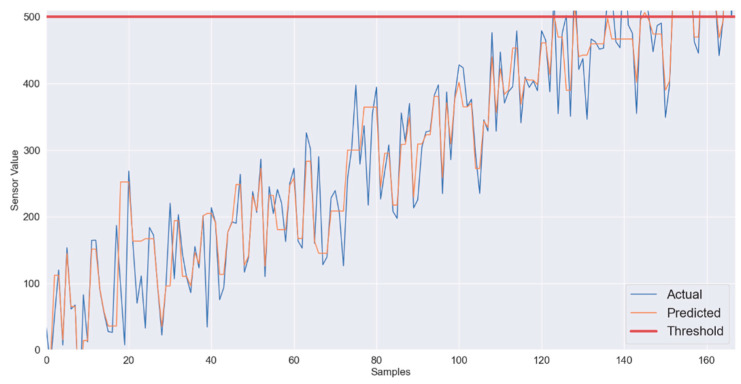
XGB regressor-average data variations: Predicted vs. Actual.

To investigate the suitability of the proposed machine learning algorithm further, rapidly changing sensory data were also considered which are presented in Figure 5 and Figure 6. Here the sensory data are changing linearly as well, but the change from normal to critical takes place in only 250 ms. In Figure 6, three series represent actual values, predicted labels and the threshold values. The mean absolute error obtained for the scenario in Figure 6 is 5.04. As shown, in Figure 6, the XGB based regression algorithm also predicts this rapidly changing sensory data very effectively.

**Figure 7:**
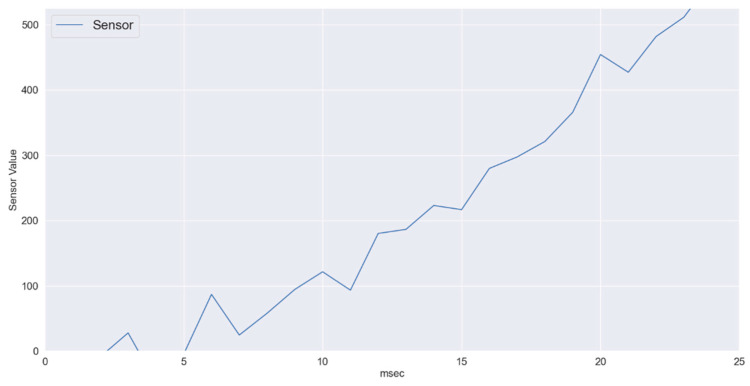
Rapidly changing sensory data.

**Figure 8:**
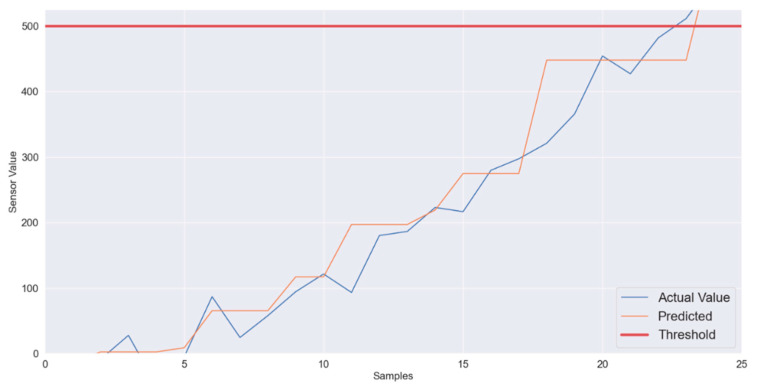
XGB-rapid data variations: Predicted vs. Actual.

Finally, the exponentially changing sensory data are evaluated as presented in Figure 7 and Figure 8. While the fluctuation in the sensory data providing a patient’s vitals changes rapidly from normal to critical, the XGB still manages to predict the data effectively. The MAE obtained between the actual and predicted values (using XGB) for the scenario in Figure 8 is 15.53. Since the sensory data change is simulated to be exponential in nature, the change in sensory data from normal to critical only takes around 22 ms. However, as shown in Figure 8, the predicted results are still very close to the actual values, thus enabling an accurate prediction of a patient’s sensory data fluctuation. This not only enables early prediction of any change in a patient’s health and vitals but also allows intermittent service failure of IoT networks to be overcome. The evaluation of results for slow as well as rapidly changing data also reveals that it is possible with the proposed regression algorithm to track the changes accurately.

## 5. Conclusions

This study developed a novel IoT and AI enabled solution to support remote monitoring of critical and non-critical surgery patients. The proposed IoT based system for healthcare applications offered ultra-reliable low latency communications (URLLC). In comparison to IEEE LLDN, the proposed IoT based solution reduced the overall delay up to 80%. In addition, the average delay was also kept within the desired limits for critical healthcare patients. The overall packet reception rate was also improved in the proposed work as compared to IEEE LLDN. The findings also suggest that the proposed XGB based regression is capable of effectively monitoring and predicting the patients’ sensory data (patients’ vital signs) in a normal as well as a critical situation. The results show that the XGB based regression is capable of predicting high sensory data fluctuations (normal to critical) within a short time window (<25 ms). Collectively, the proposed work also offers the potential to address the challenge of intermittent interference causing communication failures in short time frames, thus making the proposed system highly reliable.

The study comes with several limitations. The vital signs’ sensory dataset is generated synthetic, and it would be interesting to see how the proposed IoT based model fits the real-world monitoring of patients when collected in the clinical setting. The proposed machine learning based vital signs detection in post-surgery patient monitoring is not comparable with any previous studies as none of the existing works has developed the scenario presented in this study to monitor postoperative patients in clinical settings through vital sensory traces and clinical measures. Future work should focus on the real-world implementation of the proposed IoT framework and to explore vital physiological sensory measures such as the electrocardiogram, electroencephalogram, respiratory sensors, pulse oximetry, etc.

As a future prospect, this work can further be extended by interlinking the machine learning based analysis of patients’ vitals to evaluate the criticality index, thus, providing an extensive support system for condition monitoring and health analysis of surgery patients.

## Figures and Tables

**Table 1:** Description of frequently used variables in the proposed IoT framework.

**Parameters**	Variables	Value
Total nodes in a cluster	w	20–80
High Priority Nodes	m	20, 40, 60
Time slots in a superframe	n	20
Total High Priority Channels (HPC)	$R_{s}$	1, 2, 3
Total Monitoring Communications Channels (MCC)	$M_{s}$	1, 2, 3
Superframe Duration	T	10 ms
Timeslot Duration	t	~60 µs
Maximum cluster-size	$c_{m}$	80
Probability of failed communication of a node	p	0–0.25
$\mathrm{Node}\;\mathrm{communication}\;\mathrm{failure}\;\mathrm{probability}\;\mathrm{for}\;\mathrm{channel}\;f_{C}\left(i\right)$	$p_{i}$	
Control Channel	$Ch_{control}$	1
Communications/data Channel	$Ch_{data}$	1−k
Frequency channel space	$F_{c}$	-
Number of frequency channels used	k	4
Average delay variation from specified interval in IEEE low latency deterministic networks (LLDN)	$d_{LLDN}$	-
Average delay variation from specified interval in proposed scheme	$d_{RCC}$	-
Radio Communication channels	RCC_a_	
Channel Number	a	

## Data Availability

The datasets are not publicly available.
